# *Vibrio vulnificus* infection from insect bites in Shanghai: a case report

**DOI:** 10.3389/fmed.2024.1419074

**Published:** 2024-06-12

**Authors:** Xiaojie Hu, Huafa Que

**Affiliations:** ^1^Department of Traditional Chinese Surgery, Longhua Hospital, Shanghai University of Traditional Chinese Medicine, Shanghai, China; ^2^Longhua Clinical Medical College of Shanghai University of Traditional Chinese Medicine, Shanghai, China

**Keywords:** *Vibrio vulnificus*, insect, coastal city, case report, Shanghai

## Abstract

**Background:**

Infection with *Vibrio vulnificus* is associated with high rates of amputation and mortality. Alterations in the global climate have heightened the risk of atypical infections caused by this pathogen.

**Case presentation:**

In the case report we describe, a 75-year-old man residing in a coastal city contracted *Vibrio vulnificus* secondary to an insect bite.

**Discussion and conclusion:**

This case underscores the importance for clinicians of recognizing that early administration of appropriate antibiotics in patients with non-traditional routes of *Vibrio vulnificus* infection can significantly reduce rates of amputation and mortality.

## Background

*Vibrio vulnificus*, a Gram-negative bacterium, inhabits brackish waters in warm coastal regions. Infections typically arise from consuming raw or undercooked seafood or through exposure of open wounds to contaminated seawater or seafood (1, 2). *Vibrio vulnificus* is a highly pathogenic bacterium associated with a mortality rate of up to 33% (1). The mortality rate for wound infections caused by *Vibrio vulnificus* can reach 18% (3). In addition to classical infection pathways, clinicians often overlook atypical routes of transmission in clinical practice. Increasing evidence suggests that under conditions of high emissions and global warming, the endemic range of *Vibrio vulnificus* is expanding annually. Moreover, the role of terrestrial animals, plants, and insects as vectors is likely to elevate the risk of infection (4, 5). This case serves as a crucial reminder for clinicians to remain vigilant regarding atypical transmission routes of *Vibrio vulnificus* and provides evidence supporting insect-mediated transmission of the bacterium.

## Case presentation

The patient is a 75-year-old male who resides in Shanghai, China, approximately 70 km from the nearest coastline. His medical history includes hypertension, which has been managed with long-term medication.

**Figure 1:**
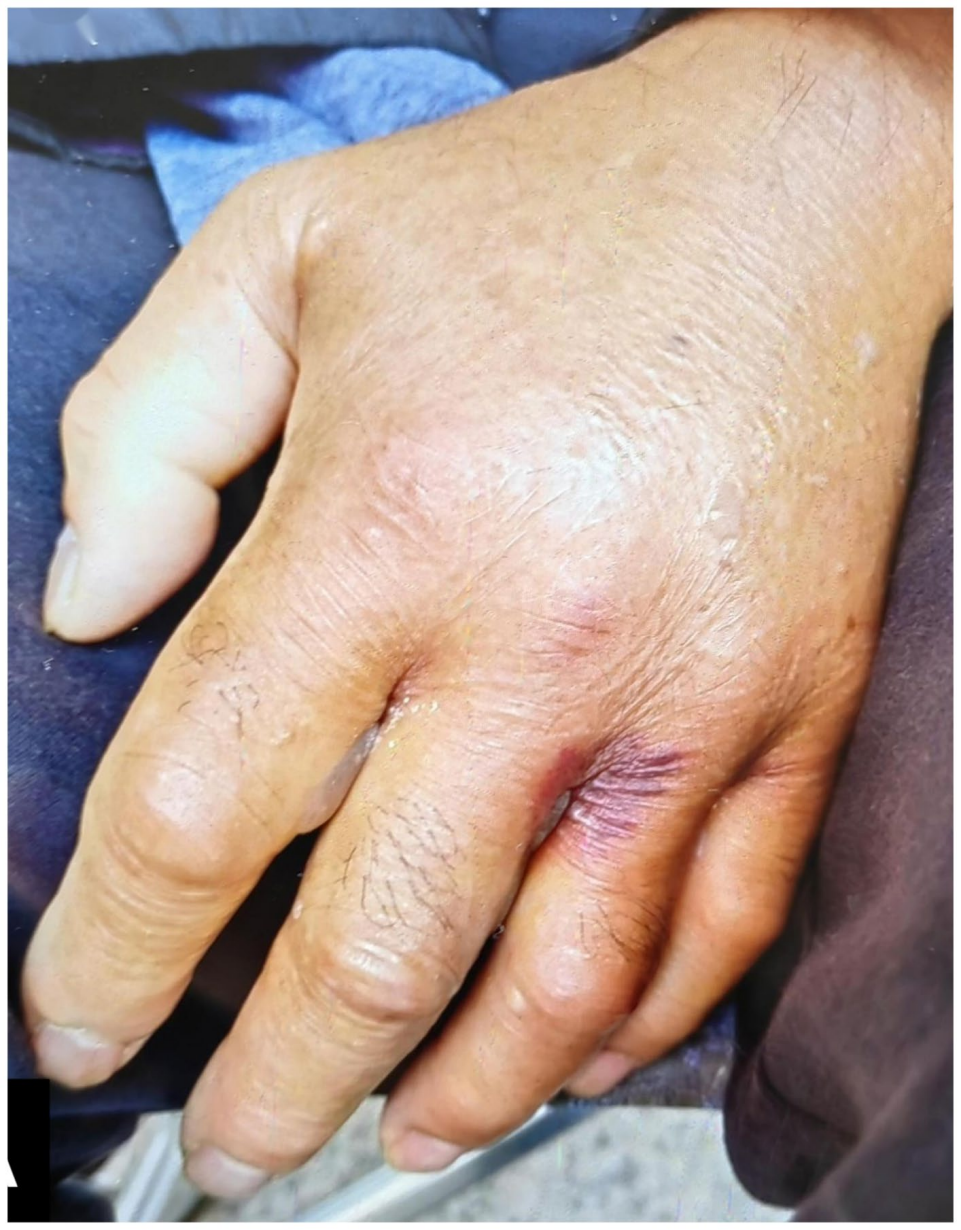
28 h after being bitten.

On the morning of September 17, 2022, while taking a walk in his community, the patient sustained an insect bite on the middle finger of his left hand. The bite, small with a diameter of 2–3 mm, caused only minor stinging pain and no other immediate discomfort. The patient disinfected the bite site but did not cover it with a waterproof bandage. The wound scabbed over within a few hours. In interviews, he and his family denied any exposure to raw fish, seawater, uncooked seafood or its juices, marine-related products, or fishing activities, as well as the use of water that might have been contaminated. No similar symptoms were reported by others in his vicinity. Nineteen hours post-injury, the patient experienced localized pain in his left middle finger, along with swelling and redness of the hand. He sought treatment at a local hospital, where he received an intramuscular injection of dexamethasone and was prescribed cefaclor capsules, olopatadine hydrochloride, and topical halomethasone cream. Despite these interventions, his symptoms persisted and the condition worsened. Twenty-four hours after the injury, the affected skin darkened, and purple blisters formed (Figure 1). Twenty-eight hours post-injury, the patient presented at our hospital’s emergency department with symptoms consistent with severe inflammation. Notably, transparent blisters had formed between the second and third fingers of the left hand, and the left upper extremity exhibited pronounced erythema and pain. The patient’s body temperature was elevated at 38.5°C, and he reported dizziness. Upon admission, laboratory findings revealed a white blood cell count of 10.48 × 10^9/L and a B-type natriuretic peptide level of 1,427 pg/mL; however, hepatic and renal functions remained within normal ranges.

Based on the findings, the physician diagnosed gangrene accompanied by insect bites on the left upper extremity. Treatment included administration of fosfomycin for its antimicrobial properties, low molecular weight heparin calcium to prevent thrombosis, and agents to protect the gastric mucosa. Concurrently, fluid from the blister was aspirated and submitted for microbiological analysis. On the first day of hospitalization, the microbiology laboratory detected *Vibrio vulnificus* in the submitted sample using microbial protein analysis techniques, with the finding confirmed by a second test. Based on the antimicrobial susceptibility report, the medication regimen was adjusted to include targeted antibiotics. During the hospital stay, the patient underwent two decompression surgeries on the skin and subcutaneous tissue of the left hand, with daily dressing changes and concurrent antibiotic and symptomatic treatment. The patient experienced pain in the left hand during these dressing changes, but no other symptoms of discomfort were reported. Subsequently, at the patient’s request, treatment was continued at another hospital.

## Discussion and conclusion

Given that the initial presentation of hemorrhagic blisters coincided with the site of an insect bite, we hypothesize that the insect bite facilitated the patient’s infection with *Vibrio vulnificus*.

*Vibrio vulnificus*, a naturally occurring Gram-negative bacterium, is commonly found in warm, brackish waters with low salinity across the globe (6). *Vibrio vulnificus* infection can manifest within 4 h, with untreated cases potentially resulting in a mortality rate of up to 100% within 3 days (7). Individuals over the age of 40, particularly men, are predominantly affected by *Vibrio vulnificus* infections (8). These infections typically manifest as acute fever, chills, hemorrhagic bullae, and septic shock.

*Vibrio vulnificus*, traditionally associated with seafood, is increasingly being reported in freshwater contexts. Exposure to freshwater fauna, flora, and insects has been identified as a contributing factor to the rising incidence of infection (9, 10). Consequently, clinicians must maintain vigilance for non-traditional routes of infection. Early administration of appropriate antibiotics is crucial to reduce the rates of amputation and mortality.

## Data availability statement

The original contributions presented in the study are included in the article/supplementary material, further inquiries can be directed to the corresponding author.

## Ethics statement

Ethical approval was not required for the studies involving humans because this article is a descriptive case report with no interventional treatment and no ethical issues involved. The studies were conducted in accordance with the local legislation and institutional requirements. The participants provided their written informed consent to participate in this study. Written informed consent was obtained from the individual(s) for the publication of any potentially identifiable images or data included in this article.

## Author contributions

XH: Writing – original draft. HQ: Visualization, Writing – review & editing.
